# Neuronal nicotinic alpha7 receptors modulate early neutrophil infiltration to sites of skin inflammation

**DOI:** 10.1186/1742-2094-7-38

**Published:** 2010-07-12

**Authors:** Lorise C Gahring, Amber V Osborne, Michelle Reed, Scott W Rogers

**Affiliations:** 1 Geriatric Research, Education and Clinical Center, Salt Lake City VA Medical Center, USA; 2 Division of Geriatrics, Department of Internal Medicine, University of Utah School of Medicine, Salt Lake City, UT, USA; 3 Department of Pathology, University of Utah School of Medicine, Salt Lake City, UT, USA; 4 Department of Neurobiology and Anatomy, University of Utah School of Medicine, Salt Lake City, UT, USA

## Abstract

**Background:**

A major site of initiation of inflammatory responses upon physical perturbation(s) and infection by invading organisms is the skin. Control of responses in this organ is, in part, modulated by the neuronal nicotinic acetylcholine receptor (nAChR) alpha7.

**Methods:**

To further investigate the role of alpha7 in skin inflammatory responses, a local inflammatory response was induced by topical application of croton oil to the ear skin of wild-type (alpha7WT) and alpha7 knock-out (alpha7KO) mice. Cells infiltrating the inflamed tissue were characterized by flow cytometry and RNA analysis.

**Results:**

Six hours following croton oil application, analysis of infiltrating cells showed that the alpha7KO mice exhibited a significantly enhanced number of cells, and specifically, of Ly6G positive neutrophils. Macrophage and lymphocyte infiltration was equivalent in the alpha7KO and alpha7WT mice. RNA analysis showed that IL-1β and IL-6 were increased significantly in the infiltrating cells of the alpha7KO mouse, although TNF failed to reach significance. In contrast, resident cells of the skin exhibited no differences in these cytokines between genotypes. Both resident and infiltrating cell populations from alpha7KO mice did show elevated message levels for the adhesion protein ICAM1. Measurement of chemokines revealed enhanced expression of the skin-related CCL27 by resident cells in alpha7KO mice. Further, we demonstrate that the population of Ly6G^+ ^neutrophils at the croton oil-inflamed skin site expresses low levels of CCR10, a receptor for CCL27 normally associated with lymphocytes.

**Conclusion:**

nAChRalpha7 in the skin can impact on early local inflammatory responses mediated through a novel population of neutrophils that are Ly6G^+^CCR10^lo^.

## Background

Neuronal nicotinic acetylcholine receptors (nAChR) are well characterized for their role(s) in modulating neurotransmission including the addictive response to nicotine [1,2]. However, these ligand-activated ionotropic receptors, particularly the distinct subtype termed alpha7 (α7), also participate in non-neuronal peripheral systems to impact upon pro-inflammatory responses [3-6]. Not unlike α7 in the nervous system, the effects of α7 activity peripherally can vary greatly depending upon where it is expressed and in which cell type. In neurons, the α7 receptor antagonizes TNFα-induced neuroprotection in primary cultures; and in peripheral organs receiving vagal innervation, the α7 receptor impacts upon inflammatory responses [4,7]. A robust contribution by α7 towards modulating inflammatory cytokine production in the skin is also present and occurs independently of vagal-parasympathetic innervation (e.g. see discussion in [3,8]). Keratinocytes appear to be the primary source of acetylcholine (ACh) in the skin [4] but further investigation regarding other cell sources is needed.

The skin is a first line of defense against many pathogenic agents and is exposed to many external agents that are pro-inflammatory (e.g., UVB or sunburn and topical irritants) as well as to agents that modify α7 function such as nicotine. Almost 30 years ago it was recognized that nicotine could alter wound healing in rabbit ears [9]. Since then, it has been further demonstrated that keratinocytes express nicotinic receptors and modulation in wound healing can be mediated, in part, through nicotinic receptors α3, α4, α5 and α7 [10-13]. Keratinocyte expression of α7 has been demonstrated using *in situ *hybridization, double-labeling immunofluorescence, and patch-clamp studies [14,15], and respond to nicotine. In tissue culture systems, Grando et al. [16] have demonstrated that nicotine increases cell-cell adherence of cultured keratinocytes and stimulates their lateral migration. The data presented here will demonstrate that α7 can also modulate the inflammatory response in skin that has been treated with croton oil.

Chemokines are another component of the inflammatory process that are crucial for the development of the appropriate response in specific tissues. This includes the skin, which has a distinct expression pattern of chemokines and their receptors (e.g., [17-19]). Chemokines are secreted basic proteins that bind to glycosaminoglycans on cell surfaces or the extracellular matrix [19,20]. Therefore, chemokines can be released into the circulation but also remain concentrated at specific sites of injury. Leukocytes in the bloodstream establish a loose tethering interaction with the endothelium which is mediated by selectin and integrin molecules such as E-selectin and α4 and β2 integrins [20,21]. This initial interaction slows the responding cell (e.g., neutrophils and lymphocytes) down and allows it to roll along the endothelium where it interacts with other cell surface receptors including chemokine receptors. The adhesion of leukocytes to the endothelium then induces intracellular signaling cascades and leads to cellular reorganization and migration of the leukocyte into the target tissue [19,20]. Once inside the target tissue, the leukocyte will release inflammatory mediators where they in turn carry out specific functions.

This study continues the investigation of how nAChRα7 influences the inflammatory response through measurements of pro-inflammatory cell recruitment induced by local inflammation in the skin. To do this, croton oil was topically applied to the ear and infiltrating cells were isolated and characterized using flow cytometry. nAChRα7KO mice exhibited significant increases of Ly6G positive neutrophils relative to controls (α7WT) at the site of inflammation. RNA analysis of both infiltrating and resident cells revealed increased IL-1β and IL-6 (but not TNFα) in α7KO infiltrates as well as certain adhesion proteins such as ICAM1 (especially by resident cells). There was also an increase in the expression of the CCR10 chemokine receptor by infiltrating cells, as wells as an increase in the ligand for this receptor, CCL27, by resident cells. Because these chemokines are important mediators of pro-inflammatory cell recruitment to the skin, our results advance the idea that α7 influences inflammatory processes to include modulation of key chemokine signaling pathways.

## Methods

### Animals

These studies were IACUC approved. All mice were housed in a pathogen free environment with water and standard mouse chow provided ad libitum. Each experiment used groups of 3-5 mice that were age (3 to 6 months old), gender and strain matched. Wild-type (WT) and α7KO mice in the C57BL/6 background were initially purchased from Jackson Laboratories (Bar Harbor, ME) and subsequently maintained as a breeding colony in our animal facility. The α7KO and α7WT mice are routinely generated by breeding mice that are α7WT/KO heterozygotes to provide homozygote litter-mate mice of both α7KO and α7WT genotypes. The α5 (WT and KO) and β4 (WT and KO) mice are also maintained in breeding colonies in the University of Utah Comparative Medicine Department.

### Isolation of ear infiltrates

The ears of α7WT or α7KO mice were treated with 10 μl of croton oil (Sigma, St Louis MO), that was diluted to a final concentration of 3.5% in 4:1 acetone: olive oil, on each side of their ears for a total of 20 μl per mouse ear. Inflammation was allowed to develop for 6 hrs before mice were sacrificed and the ears were collected. Inflamed ears were briefly dipped in 70% ethanol and PBS and separated with sterile forceps into dorsal and ventral layers. Each ear half was floated dermal side down overnight in RPMI media (Cellgro/Mediatech, Hernden, VA) supplemented with penicillin/streptomycin (Cellgro/Mediatech, Hernden, VA) and Hepes buffer (Cellgro/Mediatech, Hernden VA). The following day, infiltrate depleted ear halves were removed, and infiltrating cells were collected from the media. Cells were then washed with cold PBS and centrifuged at 1600 rpm, 4°C for 10 minutes. The ear tissue depleted of infiltrating cells was frozen (-80°C) and saved for RNA analysis. It should be noted that no cells were present in the media from ears of mice if an inflammatory stimulus (croton oil) was not first applied. Further, at times prior to 6 hours of croton oil exposure, minimal cells were isolated from ears making analysis of these cells difficult.

### Isolation of bone marrow and spleen cells

Marrow from femurs and tibias was extracted by cutting off the ends of the bones and flushing the shaft with ice-cold PBS using a 25-gauge needle and 3cc syringe. The resulting mixture was passed through the needle and syringe once to obtain a single-cell suspension. Cells were centrifuged at 4°C, 1600 rpm for 5 minutes, and then resuspended in erythrocyte lysis buffer (0.14 M Ammonium Chloride, 17 mM Tris in H_2_0) for 5 minutes at 25°C. Cells were washed with 10 ml ice-cold PBS, centrifuged, and collected for further analysis. Similarly, spleens were removed and cells expressed with forceps. Single cell suspensions were then treated with ammonium chloride to lyse red blood cells, washed and collected for further analysis.

### Cell cytometry analysis

To analyze cells by flow cytometry, cells were counted with a hemocytometer and 0.5 to 1 × 10^6 ^cells were resuspended in 1 ml PBS, and placed into tubes on ice. For all samples, Fc receptors (Fc block, BD Bioscience Pharmingen, San Jose, CA) were blocked using 1 μg per 10^6 ^cells for 15 minutes on ice. The effectiveness of this block was tested by subsequent staining for FcR using labeled 2.4G2 antibody which routinely showed complete block. Antibodies were added to the cell samples at a concentration of 1 μg per 10^6 ^cells and incubated for 30 minutes on ice in the dark (FITC-conjugated anti-Ly6G, anti-CD45R/B220, anti-CD4, anti-CD8α from BD Bioscience Pharmingen, San Jose, CA; PE-conjugated anti-Ly6C (BD Bioscience Pharmingen, San Jose, CA), anti-CCR10 (R&D Systems, Minneapolis, MN), anti-F4/80 from eBioscience, San Diego, CA). Isotype controls to assess non-specific binding were FITC- or PE-conjuated rat IgG2a and PE-conjugated rat IgG2b (BD Biosciences Pharmingen, San Jose, CA). Following incubation with antibodies, cells were washed with PBS and filtered through 85 micron nylon mesh (Small Parts Company, Miami Lakes, FL) to remove aggregated cells. Samples were analyzed using either a Becton Dickinson FACScan instrument or an Accuri Cell Cytometry System (Ann Arbor, Michigan). Data were analyzed with FlowJo Software (Ashland, OR). Histogram data are expressed as percentage of maximum according to standard flow cytometric analysis in which peak heights within each graph are normalized to a percentage of the peak representing the highest event number on the X axis. The highest event number is based on 'bins' which are numerical ranges for the parameter on the X axis (as described in the Flowjo.com for cell cytometry histogram generation). Data displayed as percent of Max allows for visual comparison of samples with different event numbers collected.

### RNA isolation for arrays and real-time PCR

To extract RNA, cells were placed in Trizol (Invitrogen, Carlsbad CA), and processed for RNA according to the manufacturer's instructions. Ear skin (devoid of infiltrating cells) was also placed in Trizol and homogenized with a tissue tearor (Biospec Products, Bartlesville, OK). Manufacturers' directions were then followed for RNA isolation. RNA was precipitated in isopropanol and washed in 75% ethanol at room temperature. The concentration and purity of the RNA was determined with a ND-1000 Spectrophotometer (NanoDrop Technologies, Wilmington DE). A 1 μg sample of each RNA was DNase treated and converted via reverse transcription PCR to cDNA (Promega, Madison WI) and used to amplify cDNA (high capacity cDNA Archive Kit, Applied BioSystems, Foster City CA). 20 ng of cDNA was loaded into each well and the following TaqMan Gene Expression Assays were used: ICAM1 (Mm00526023_m1), Itga3 (Mm0042890_m1), Itgb2 (Mm00434513_m1), E-Selectin (Mm00441278_m1), IL1-β (Mm00434228_m1), IL-6 (Mm00446190_m1), TNFα (Mm00443258_m1), CXCL1 (Mm00433859_m1), CXCL5 (Mm00436451_g1), CXCL9 (Mm0043494_m1), CCL1 (Mm00441236_m1), CCL2 (Mm00441242_m1), CCL3 (Mm00441258_m1), CCL4 (Mm00443111_m1), CCL5 (Mm01302427_m1), CCL11 (Mm00441238_m1), CCL27 (Mm00441257), CCL28 (Mm00445039_m1), GAPDH (4352339E), β-actin (4352341E) (Applied Biosystems, Foster City CA). GAPDH and β-actin were used as internal control genes, and all samples were run in duplicate. Fold-changes in RNA expression were calculated relative to RNA from matched control animals, and was determined using the comparative Ct method (Applied Biosystems). RNA from both skin tissue and infiltrating cells was also analyzed in duplicate using Mouse Inflammatory Cytokines and Receptors RT^2 ^Profiler PCR Arrays (SABiosciences, Mouse Inflammatory Cytokines and Receptors, PAMM-011E) according to manufacturers' directions. Briefly, RNA was converted to cDNA using the RT^2 ^First Strand Kit, and amplified using RT^2 ^SYBR green/ROX qPCR master mix (SABiosciences, Frederick, MD). WT transcript levels for each experiment were normalized to a value of 1 to allow generation of fold change values in the alpha7KO in each experiment, and to compare fold change with the KO between experiments. Error bars reflect the standard error of the mean as calculated from 3 independent experiments with 5-6 mice per experimental group.

## Results

### Infiltration of pro-inflammatory cells is modified by α7 expression

**Figure 1:**
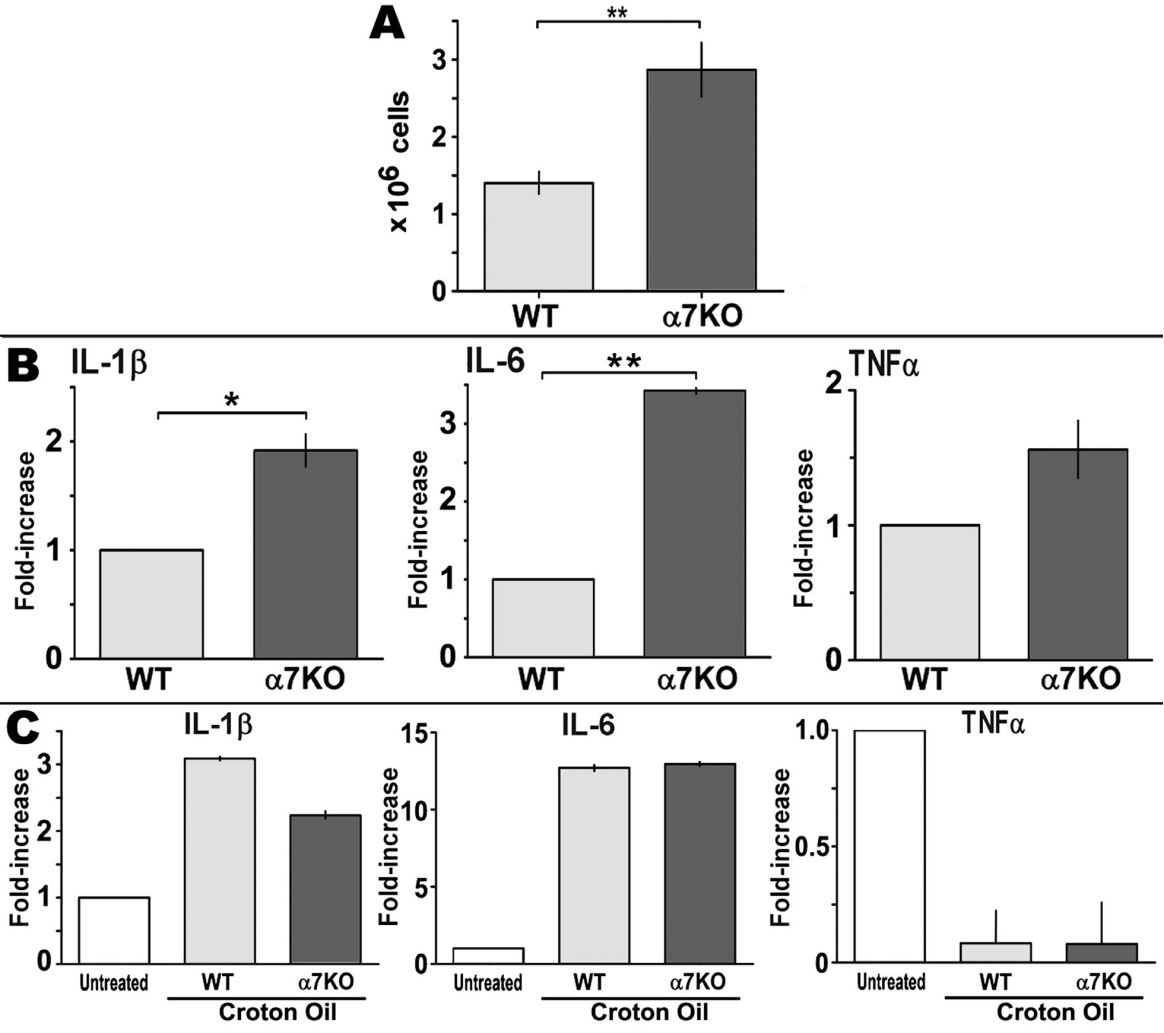
**The local inflammatory response to croton oil is enhanced in the α7KO mouse**. Ears of WT and α7KO mice (5 animals/experiment) were treated with croton oil. Six hours post-treatment ears were removed, dorsal and ventral halves separated before floating them dermis side down on media overnight. **A) **Total number of cells isolated per treatment group was then measured (99% viability, not shown). The light grey bars represent WT mice and dark grey bars the α7KO. Error bars reflect ± of the standard error of the mean of all experiments (3-5 experiments per group); * p < 0.05 and **, p < 0.01 (two-tailed Student's T-test). **B) **Expression of key pro-inflammatory cytokine transcripts from infiltrating cells using qPCR (Methods). The average fold increase normalized to a value of 1 for IL-1β, IL-6 and TNFα from α7WT mice (light grey) and α7KO (grey bars) are compared. Error bars reflect the standard error of the mean from 3 independent experiments with 5-6 mice per experimental group. **C) **Analysis of transcripts from ear skin after removal of infiltrating cells from untreated mice, or mice treated with croton oil. The expression of these cytokines does not differ between α7WT and α7KO. Light gray bars represent ear skin from WT mice and dark gray for α7KO mice. Non-shaded bars represent ear skin from untreated WT mice. Ear skin RNA amounts recovered did not differ between genotypes (not shown).

The mouse ear pinna provides a unique site to study cellular infiltration following skin inflammation. This is due to the distinct dorsal and ventral sides, each with an epidermal and dermal layer, that are separated by auricular cartilage. Upon application of an inflammatory agent, cells collect between the dorsal and ventral ear skin and infiltrate the tissue. The infiltrating cells are recovered from the media after mechanical separation of the ear into the respective halves, and overnight incubation of the ear halves in media allows cells to be released into the media. This procedure results in the isolation of recruited cells without using more disruptive interventions that can inconsistently impact upon cells more sensitive to harsh isolation conditions. To examine an inflammatory response in the skin, we used the well-characterized method of topical application of croton oil, an organic phorbol compound derived from the seeds of the *Croton tiglium *tree. A particular advantage of this method is the relatively mild inflammatory response which could facilitate detection of α7 modulatory components. Mouse ears were treated with topically applied 3.5% croton oil to induce local inflammation. At six hours post croton oil application, the mice (groups of 5 α7WT or α7KO) were sacrificed and the ears removed. The dorsal and ventral sides of the ear were separated and incubated overnight in media (see Methods). The results presented in Figure 1a show that the total number of cells isolated from α7KO mice is significantly (p < 0.01) enhanced relative to wild-type mice. Cell numbers reflect the total number of cells obtained from 5 mice in each group. The standard error of the mean is derived by averaging the number of cells obtained from each of these groups (WT or KO) from at least 5 separated experiments. In the absence of an inflammatory stimulus such as croton oil, there are too few cells to isolate and characterize. Further, we have found that the overnight incubation of the ear halves is required to obtain the infiltrating cells suggesting that the cells are not just in the edematous fluid of the ear.

To determine if infiltrating cells from α7WT and α7KO mice differ in cytokine production, quantitative real-time PCR (qPCR) of key pro-inflammatory cytokine RNAs was done using Taqman gene expression assays. The cytokines examined were previously shown to differ between WT and KO mice upon ultraviolet radiation exposure to defined skin sites [8]. There was greater IL-1β and IL-6 (p < 0.05) in the α7KO cells relative to infiltrating cells from the α7WT mouse (Figure 1b). However, other cytokine RNA expression such as for TNFα, while elevated in the α7KO infiltrates, was not statistically different between genotypes. In the ear tissue remaining after depletion of infiltrating cells the expression of IL-1β, IL-6 or TNFα RNA did not differ between α7WT and α7KO mice (Figure 1c). There was, however, a substantial decrease in TNFα in the ear skin of both the α7WT and α7KO mice treated with croton oil. Thus in skin receiving croton oil there is an overall increase in the number of cells recruited to the site of inflammation in the α7KO mouse relative to α7WT. Infiltrating cells have elevated IL-1β and IL-6 message in the α7KO compared to WT animals while the resident skin cells of the WT and KO mice express equivalent amounts of these cytokines.

### Neutrophil infiltration to the site of skin inflammation is enhanced in the α7KO mouse

**Figure 2:**
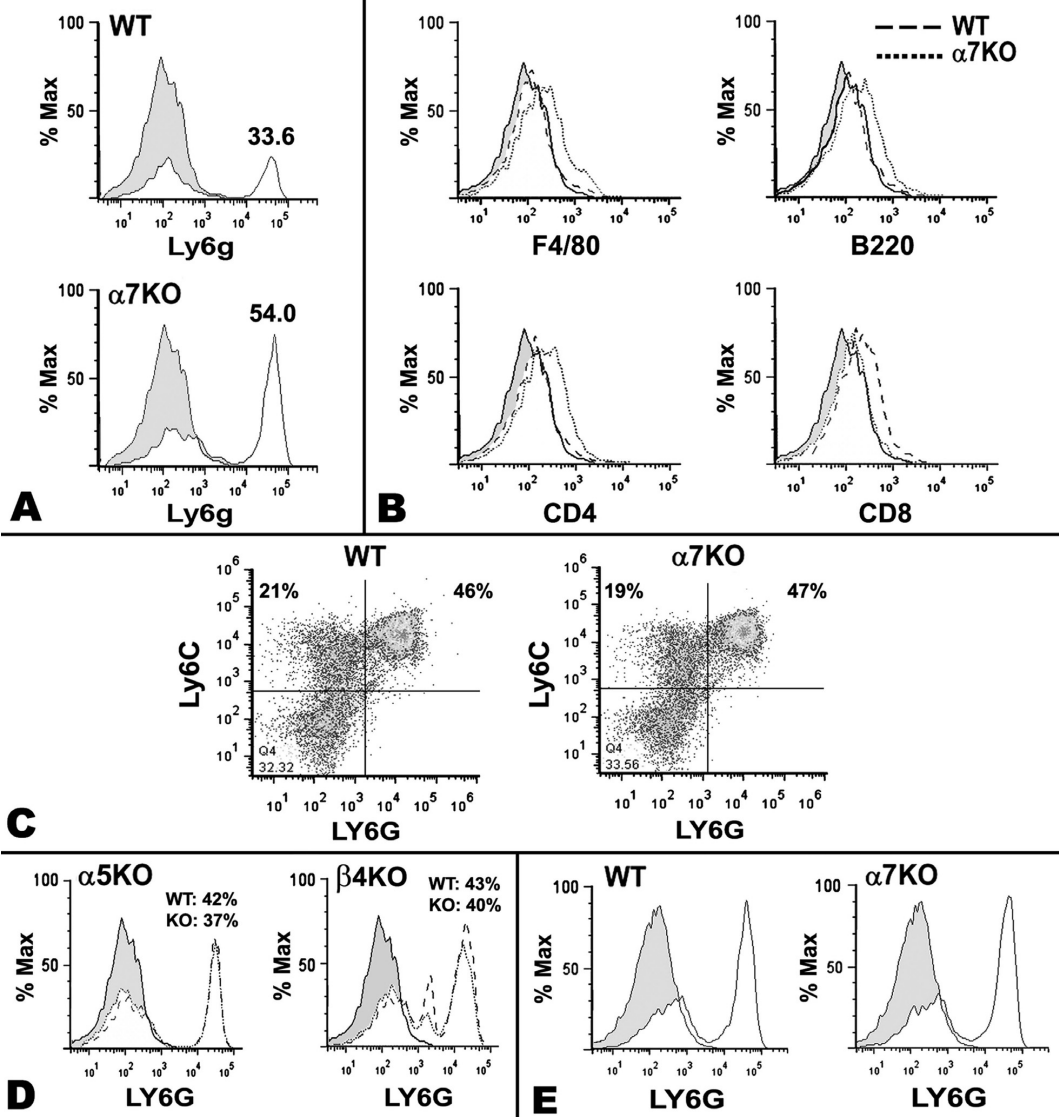
**Croton oil treatment of α7KO mice selectively enhances neutrophil infiltration into ears 6 hours post-croton oil application**. **A) **Flow cytometry of infiltrating cells from the α7WT (upper) or α7KO (lower) mice reveals a substantial difference in the infiltration by Ly6G^+ ^neutrophils 6 hours following croton oil treatment. Similar results were also obtained for C3H mice (data not shown). Histogram height is displayed as Percentage of Maximum (% Max), meaning that peak heights within each graph are normalized to a percentage of the peak representing the highest event number on the X axis. The percent of Ly6G positive cells is shown above the peak response. At least 3 independent experiments, each containing 5 mice per group, are shown and isotype controls for all antibodies showed a staining pattern identical to the unstained controls (shaded histogram). **B) **Infiltrating cells stained with other immune cell markers for α7WT and α7KO genotypes. **C) **Bone marrow granulocytes of α7WT and α7 KO mice double labeled for Ly6G and the more immature marker, Ly6C (granulocytes and neutrophils). Unlike infiltrating cells to the skin, these cells do not differ in resident bone marrow populations. **D) **Similar to Panel A, either α5KO mice or β4KO mice with corresponding WT controls were examined for Ly6G recruitment to the skin. The grey shaded histogram represents control unstained cells. **E) **Cells from ears of α7WT and α7KO mice were harvested 24 hours post application of croton oil. Results reflect similar numbers of Ly6G positive cells at this later time.

The identity of the infiltrating cells at the site of inflammation following croton oil application was determined using flow cytometry and markers specific for neutrophils (Ly6G; PMNs: polymorphonuclear cells), mature macrophages (F4/80; [22]), B-cells (B220) and T-cells (helper T-cells, CD4 or cytotoxic T-cells, CD8). As shown in Figure 2, there was a dramatic difference in infiltration by neutrophils to the site of inflammation in the α7KO when compared to the α7WT (Figure 2a). Staining with an isotype (FITC-IgG2a) control for the anti-Ly6G antibody resulted in a histogram that is identical to the unstained controls (data not shown). Results are expressed as percentage of the maximum response and percent of Ly6G positive cells is reflected by the percentage above the peak response (α7KO, 54%; α7WT 33.6 percent neutrophils). The increase in Ly6G^+ ^cells in the α7KO is also observed in α7KO mice that are in the C3H background (not shown) indicating that while B6 and C3H mouse strains may differ substantially in response to nicotine (e.g., see [2]), the results measured here are independent of mouse strain backgrounds. The increase in PMNs recruited in the α7KO mouse was consistently measured using either the anti-Ly6G antibody or anti-Ly6C antibody (which detects PMNs as well as other granulocytes, as in Figure 2c). The small difference in intensity of staining with F4/80, B220, or CD4 antibodies was not consistently observed, nor was the small decrease in intensity of staining with the anti-CD8 antibody (Figure 2b). The possibility that increased PMN recruitment in the α7KO mouse is representative of a difference in the composition of bone marrow in the α7KO mouse was examined. Bone marrow was extracted from mice of each genotype and antibodies to cell type specific markers were used to determine the percentage of neutrophils, macrophages and B cells. The results shown in Figure 2c show no difference between α7WT and α7KO mice in number of Ly6G^+ ^neutrophils or Ly6C^+ ^granulocytes or other cell types (not shown) in the bone marrow. Further, the spleen, while not a major site of neutrophil residence or source for recruitment, also showed no difference in neutrophils the between WT and KO mice.

Because nicotinic receptors such as α5 and β4 are also expressed in peripheral systems (especially in ganglia; [1]), the specificity of the influence of α7 on enhanced neutrophil infiltration was examined. As described above, croton oil was applied to ears of mice having either the nAChRα5 or nAChRβ4 subunit ablated (α5KO or β4KO, respectively). The α5KO was selected because the absence of expression by this subunit has been implicated in altering the course of other inflammatory-related diseases [23] and in some cases it has been suggested to interact with α7 to form heteromeric receptors with functional differences uniquely distinct from α7 homomeric receptors [1]. The β4 subunit is the principal beta subunit of nAChRs in peripheral nerve tissue such as autonomic ganglia [1]. Further, while mice tolerate ablation of β4, presumably due to compensation by the β2 subunit, the function of the receptor and response to external agents such as nicotine is significantly altered [24,25]. Neither subunit forms a functional receptor when expressed alone [1]. As shown for neutrophil skin infiltrates in Figure 2d, no difference was observed for either of the α5KO or the β4KO mice relative to appropriately matched WT litter-mate controls. Further, neither the α5KO nor the β4KO exhibit any other difference in cellular infiltration, such as macrophages or lymphocytes (not shown). Thus, the effects related to α7 expression are not the result of a more broad disruption of nAChRs expressed in ganglia or other peripheral sites or of non-specific genetic manipulations used to generate these mice.

To determine whether the increase in neutrophils in the inflamed ear of the α7KO mouse persists at later time points, ears were harvested 24 hours after croton oil application. Results obtained indicate that no difference in the number of neutrophils (or macrophages and lymphocyte populations, Figure 2e) was observed between the α7 WT and the α7KO mice. Thus, the absence of α7 appears to enhance the early response to an inflammatory agent while not affecting neutrophil accumulation at later times (24 hrs, discussed in the next section).

### Chemokine expression differs in cells infiltrating the skin of α7KO mice

**Figure 3:**
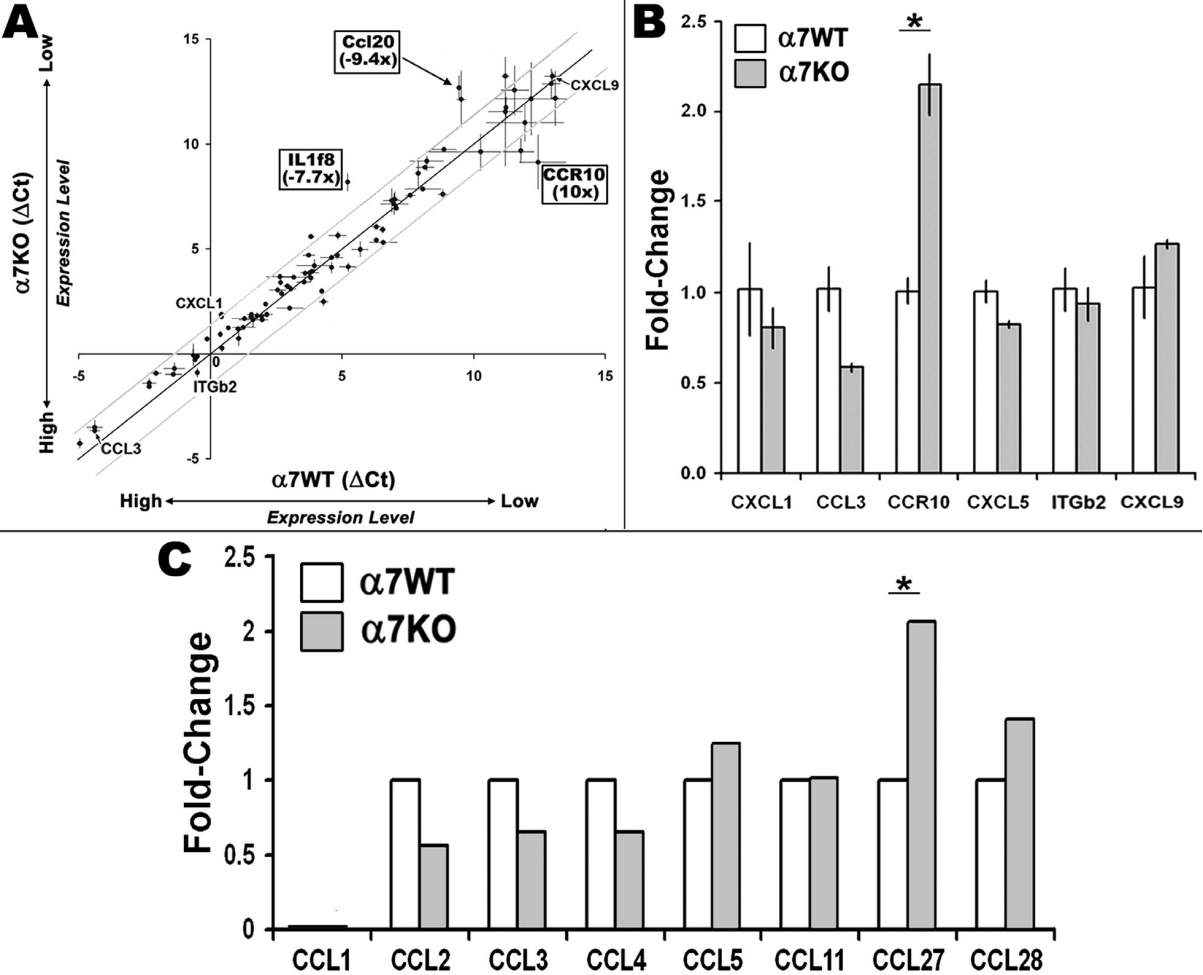
**Microarray analysis of infiltrating cells identifies the expression of the chemokine receptor CCR10 to be elevated in the α7KO**. **A) **RNA collected from infiltrating cells from croton oil treated ears was analyzed using SABiosciences arrays. As indicated, the DeltaCt (cycle number to detect transcript after normalization to (β-actin and GAPDH) is inversely related to transcript number. The central line is the linear regression best fit (R^2 ^= 0.95) and flanking dotted lines define the boundaries of statistical significance (p > 0.05). Individual error bars reflect ± the standard deviation. Transcripts that are different in expression between the α7WT and α7KO are boxed and their identity (CCR10, IL1f8 and CCl20) shown. Some additional transcripts that failed statistical significance are also identified. **B) **RT-PCR analysis (see Methods) of transcripts identified by microarray using Taqman gene expression. There is a good qualitative match with the microarray data. **C) **A similar analysis of the total ear skin depleted of infiltrating cells revealed no detectable changes in transcript expression that were confirmed by RT-PCR. Analyses for transcripts not on the array such as CCL27 and CCL28 (the ligands for CCR10) present in resident cells of the ear skin were included (* p < 0.01). Neither CCL27 nor CCL28 were detectable in infiltrating cells (not shown).

Cytokines and chemokines play a key role in normal cellular trafficking and recruitment of cells to sites of inflammation [11,19,20,26,27]. Both chemokine and chemokine receptor expression is especially important to rapid responses to assure cells are directed to the appropriate tissue sites. We determined the impact of α7 on chemokine and chemokine receptor expression by the infiltrating cell population. In the same experimental paradigm as above, infiltrating cells were harvested from ears of α7WT or α7KO mice after exposure to croton oil for 6 hours. The RNA from these cells was analyzed using commercially available microarray systems (SABiosystems, see Methods) for cytokines, chemokines and chemokine receptors. While the majority of the examined signaling molecules did not vary between mice of differing genotypes (α7WT vs. α7KO), there were prominent differences (Figure 3a). Among the most notable was the expression of the chemokine receptor CCR10 where expression was much greater in the α7KO infiltrating cells. This result was verified using qPCR (Applied Biosciences TaqMan primers) as shown in Figure 3b. CCR10 message was not detected in the skin of either the α7WT or α7KO mouse (not shown).

Although signaling through the CCR10 chemokine receptor is most often associated with lymphocytes rather than neutrophils (discussed in next section), the possibility that the expression of the ligand for this receptor was also dysregulated was examined. This was done independently of the arrays since the chemokine ligands for CCR10, CCL27 and CCL28, were not included in the arrays. Therefore, we measured CCL27 and CCL28 in RNA from both infiltrating cells and resident skin of the inflamed ear using TaqMan probes and qPCR. Neither CCL27 nor CCL28 were detected in the RNA from infiltrating cells of either the α7WT or α7KO mice (not shown). This was not unexpected since the expression of these ligands would be anticipated to be expressed by resident cells at the site of the inflammatory stimulation in order to recruit inflammatory cells. CCL27 in resident cell RNA was detected and its expression was elevated in the croton-oil exposed skin of the α7KO compared to the α7WT mouse (Figure 3c).

### Identity of CCR10 positive infiltrating cells

**Figure 4:**
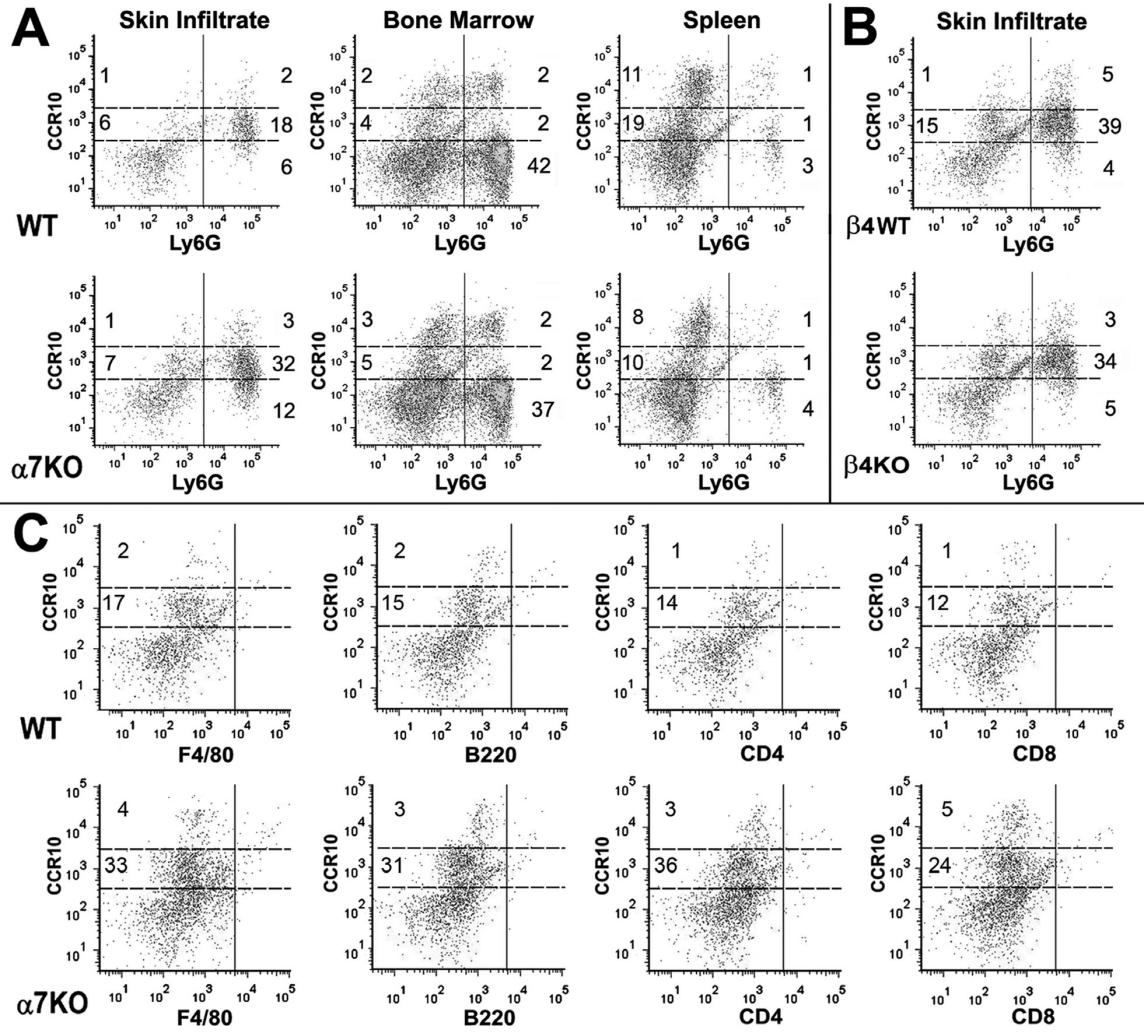
**Ly6G^+ ^infiltrating cells co-express CCR10**. **A) **Ly6G^+^/CCR10^+ ^infiltrating cells 6 hours after croton oil. Ly6G cells from the skin express low levels of CCR10 (CCR10^lo^). There are more Ly6G^+^CCR10^lo ^cells present in the α7KO (32%) versus the WT (18%). The numbers reflect percentage of cells rounded to the whole number. Analysis of bone marrow or spleen reveal no difference in neutrophil numbers relative to genotype as reported [28,30]. Isotype controls were identical to unstained controls (not shown). **B) **The percentage of Ly6G^+^CCR10^lo ^cells elicited by croton oil application to the β4WT or β4KO mice was equivalent. **C) **Croton oil induced infiltrating cells were also examined for co-expression of CCR10 with other immune cell markers including macrophages (F4/80), B-cells (B220) and T-cells (CD4 or CD8).

Because CCR10 expression is most often associated with lymphocytes [28-30], the identity of CCR10 expressing cells in the cellular infiltrates of croton oil treated mice was examined. To determine if the infiltrating neutrophils described above were CCR10 positive, cells isolated from animals (α7WT and α7KO) were co-labeled for Ly6G and CCR10. As measured using flow cytometry, Ly6G^+ ^infiltrating cells were substantially increased in α7KO mice (47% in the α7KO vs. 26% in α7WT, Figure 4a). Further, this difference was also reflected in the number of Ly6G^+ ^cells expressing CCR10 (32% in α7KO vs. 18% in α7WT). However, the intensity of CCR10 staining of these neutrophils is less than that observed for CCR10 positive cells of the bone marrow or spleen (Figure 4a). We refer to the skin associated infiltrating neutrophils as Ly6G^+^CCR10^lo^. Results in Figure 4a also show that the majority of bone marrow cells that are Ly6G^+ ^are not CCR10 positive (but Ly6G^+^CCR10^+ ^cells are definitely present) and the majority of CCR10^+ ^cells in the spleen are not Ly6G^+^, but there is a large population of Ly6G^neg^CCR10^hi^cells. Differences in the number of CCR10 positive cells between α7WT and α7KO in the bone marrow or spleen were not observed. Therefore, these results suggest a novel population of CCR10^lo ^neutrophils that are recruited to the skin. The β4KO skin infiltrate cells were also examined for the presence of Ly6G^+^CCR10^lo ^cells (Figure 4b). The results demonstrate that Ly6G^+^CCR10^lo ^cells constitute the majority of the infiltrating population of cells but no difference between the β4WT and β4KO in percentage of these cells is observed. This result again points to the specificity of the enhanced response in the α7KO mouse.

Finally, we tested whether other immune cells infiltrating the skin were CCR10 positive in the α7KO mouse. For these measurements, cells (including T-cells, B-cells and macrophages) collected from ears 6 hours post-inflammatory challenge were co-labeled with CCR10 and quantitated using flow cytometry analysis. As shown in Figure 4c, the difference between the α7KO and α7WT mice in CCR10^lo ^cells is present (as shown in Figure 4a) and these CCR10^lo ^staining cells are F480^neg^B220^neg^CD4^neg^CD8^neg^. Thus, croton oil challenge to the α7KO mouse results in a greater number of Ly6G^+^CCR10^lo^, as compared to the α7WT mouse, at the site of inflammation.

### Other factors affecting cellular trafficking

**Figure 5:**
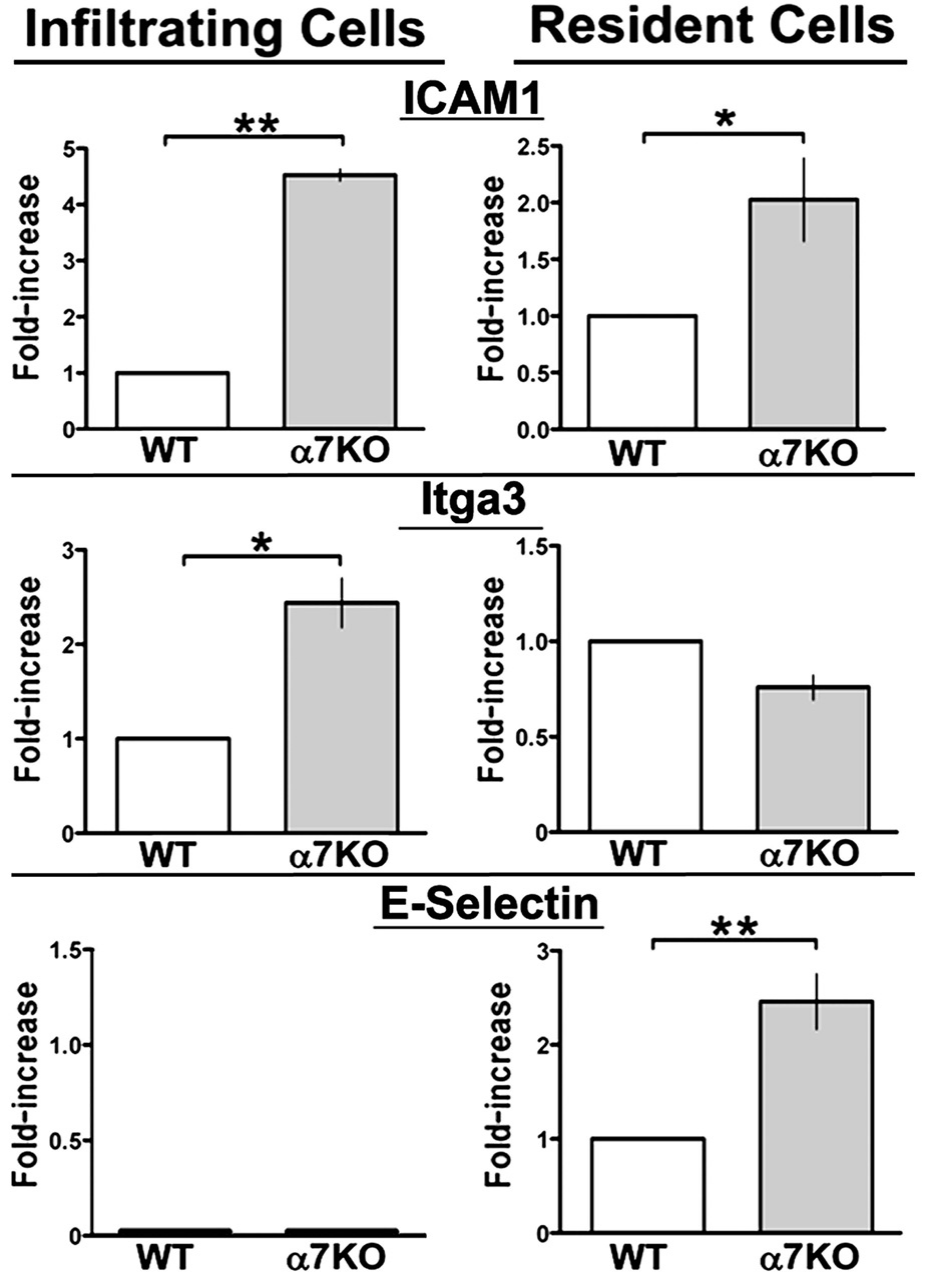
**The expression of adhesion molecule transcripts is altered by α7 expression**. Examination of infiltrating cells and resident cells after croton oil treatment for 6 hours reveals that certain molecules important to cell recruitment and localization to inflamed tissue are expressed. As shown using qPCR, in α7KO mice transcripts for ICAM1 are enhanced considerably in infiltrating cells and to a lesser extent in resident cells relative to controls (WT mice values normalized to 1 were used to determine fold increase observed in the KO). Itga3 transcripts are increased in α7KO infiltrating cells. In contrast, E-selectin transcripts are elevated in resident cells (ear skin depleted of infiltrating cells) and especially in the α7KO, but these transcripts were not detected in infiltrating cells. Error bars represent standard error of the mean; * p < 0.05; ** p < 0.01.

Integrins and adhesion proteins also play a central role in the proper trafficking and accumulation of cells at sites of inflammation. The change in transcript expression of some adhesion proteins including ICAM1, integrins α3 (Itga3) and β2 (Itgb2), and E-selectin were measured and are shown in Figure 5. The results show that E-selection (CD62E), which is normally present on endothelial cells, is increased in the skin (resident cells) of the α7KO mouse relative to the α7WT animal (Figure 5, bottom panel) and is not detectable in the infiltrating cells (not shown). In infiltrating cells the α3 integrin Itga3 was elevated in the α7KO mouse but not in the resident tissue of α7KO mice where expression did not differ from the α7WT. The β2 integrin expression did not differ between α7WT and α7KO (not shown). ICAM-1 (CD54), which can be expressed on a variety of cells including hematopoietic cells and resident cells such as fibroblasts and keratinocytes, is elevated in both the infiltrating cells and the resident tissue of the α7KO mouse. Therefore, selective differences in the expression of adhesion molecules and chemokines are observed in the α7KO mouse that could account for the increase in PMN recruitment and accumulation to sites of inflammation.

## Discussion

The skin is a site of frequent but localized pro-inflammatory events. The rapid onset of local inflammatory processes includes elevated cytokine and chemokine production as well as cellular infiltration. Nicotinic acetylcholine receptors (nAChR), while best characterized as neurotransmitter receptors on neurons, also contribute to host defenses in many tissues, including the skin, through mechanisms not necessarily associated with the more recognized role in modulating neurotransmission [1-3,6,31]. A prominent nAChR composed of the α7 subunit, as well as its endogenous agonists acetylcholine and choline, are expressed by keratinocytes [6,32,33]. The status of acetylcholine production by cells in the skin, other than keratinocytes and especially in the context of the inflammatory response, remains to be fully explored. The nAChRs are also responsive to the exogenous agonist, nicotine. Much of what we know about the role of this receptor in inflammation has been found through examining cultured cell systems or the mouse model (especially mice lacking α7 expression, α7KO). Many studies have demonstrated that in the absence of α7, the response to inflammatory stimuli is elevated. This includes the influence of α7 on inflammatory pro-inflammatory cytokine signaling (e.g., TNFα [7]), damped inflammatory cytokine expression by macrophages (see [34] but also see [35]), altered subsequent adaptive immune responses including antibody production [36], and shifts in inflammatory responses in tissues receiving vagal nerve innervation [34,37]. In the lung, an influence by α7 on acid-induced acute lung injury in the mouse model is observed and includes elicitation of more inflammatory cytokines in the α7KO over the α7WT animals [38]. This increase in lung inflammatory cytokines in the α7KO mice is associated with more severe lung injury than the WT mice suggesting that this receptor plays a regulatory role in balancing the inflammatory processes in the lung. In a previous report [8] we demonstrated that α7KO mice elicit a greater response to a common environmental inflammatory agent, ultraviolet radiation (UVR). The skin does not receive significant parasympathetic or any vagal innervation indicating that these neuronal systems are not directly required for α7 to impact on these processes. A more local control of signaling is also supported by studies examining keratinocytes cultured *in vitro *which express nAChRs, including α7, and these impact upon multiple cellular processes including cell migration and the expression of various chemokines and adhesion molecules [39-43]. Thus, it can be hypothesized that the role of α7 in normal inflammatory responses in the skin are likely responsive to acetylcholine or choline and suggest that the normal physiologic function of this nAChR in regulating inflammation is customized according to tissue, cell type and neuronal complexity.

In this report the lack of α7 was found to modifying cellular infiltration to a local inflammatory stimulus of the skin. As reported previously, the lack of α7 expression (α7KO mice) corresponds with modulation of pro-inflammatory molecules such as IL-1β and IL-6 [7,8,44]. We now find this includes an early enhancement of neutrophil infiltration to an inflamed area of the skin that is not due to increased numbers of available neutrophils in the bone marrow or spleen of the α7KO mouse. Consistent with altered recruitment of neutrophils is a coincident increase in expression by the α7KO mouse of the skin-associated signaling chemokine, CCL27, and its receptor (CCR10) that is expressed by responding infiltrating cells. This is significant in terms of identifying potential specific contributors in these interactions. The chemokine system, consisting of four gene families distinguished by the position of their cysteine residues [19,20], is complicated by the considerable promiscuity in ligand-receptor interactions and tissue diversity in expression (for detailed review see [19]). In general, lymphocytes are reported to express predominantly CCR receptors that interact with CC chemokines. In contrast, neutrophils express predominantly CXCR receptors that interact with the CXC chemokines. Recruitment of cells to the skin through elicitation by CCL27 is not unexpected since this chemokine is known to be induced by specific skin-inflammatory cell interactions [28-30]. However, CCR10 expression by neutrophils has to our knowledge not been reported. Our results find that the neutrophils, as defined by Ly6G (and co-expression of Ly6C) expression, are recruited in both the α7WT and the α7KO mouse and these cells also exhibit CCR10 expression. The skin infiltrating cells from the α7KO mice also express elevated RNA transcripts for this chemokine receptor. Notably, the majority of the infiltrating Ly6G^+ ^neutrophils were CCR10 positive but stained less intensely for this marker than neutrophils in the bone marrow or spleen. Further, in the bone marrow and spleen the majority of CCR10 positive cells are not Ly6G^+^. The significance of this population of neutrophils in the skin and their characterization as Ly6G^+^CCR10^lo ^remains to be investigated further. A recent report [45] showing that α7KO mice elicit an accelerated clearance of *E. coli *injected into the peritoneum of mice through a more rapid recruitment of neutrophils to the site of infection suggests that these findings could be more generalized to include sites other than the skin. However, it is noteworthy that the impact by α7KO on bacterial clearance was transient during the early phase of bacterial infection [45]. Our results are consistent with the role of α7 being most important early in an inflammatory response where it appears to limit (or in the case of the α7KO enhance) the magnitude of initiating events. This in turn would correspond with control of greater tissue damage as has been suggested from studies of lung responses in α7KO mice [38] and our report that the skin of the α7KO mouse demonstrates greater edema and thickness following ultraviolet radiation of back skin [8].

Another alteration in α7KO mice is a change in the expression of adhesion molecules in both the target tissue and the trafficking cells. The specificity of the expression of adhesion molecules by either infiltrating cells (e.g., Itga3 in Figure 5) or resident cells (e.g., E-selectin Figure 5) is also notable. This suggests that α7 expression in the skin influences multiple mechanisms downstream of receptor activation including shifts in cytokines and chemokines that will subsequently have a broad effect on many cells in the environment regardless of their α7 expression status.

The presence of sustained nicotine exposure, which impacts all nAChRs, would likely introduce an imbalance to cellular pathways, of which α7 is a modulatory component. Similar to neurological systems where imbalance in nAChR expression leads to phenotypes of addiction [1], the interaction between nAChRs and pro-inflammatory systems is also finely balanced. For example, it has been reported that acne and eczema are more common in nonsmokers suggesting that nicotine may actually dampen specific inflammatory responses in the skin leading to these diseases [46,47]; Or, such responses may reflect an altered 'tone', perhaps demonstrated by elevated baseline levels of inflammatory cytokines. This is not inconsistent with the calming effects of nicotine on the inflammatory bowel disease ulcerative colitis (UC) and where cessation of smoking correlates with an induced flare in this disease (see [3]). However, nicotine exacerbates Crohns disease [48-52], a related inflammatory bowel disease of different pathology. That the active compound is nicotine has been demonstrated many times with delivery of this compound through patches placed on rats, mice and in some cases humans and appears to reverse some inflammatory symptoms while exacerbating others [53,54]. These interesting associations again point to the fact that we have much to learn about nAChR regulation of inflammation in different tissues whose mechanism(s) reflect a summation of the impact by multiple nAChRs and intracellular signaling systems. Further, smoking is associated with premature aging of skin which may be a result of nicotine effects on the normal responses of the nicotinic receptors in this tissue.

## Conclusion

This study finds that mice lacking nAChRα7 have elevated levels of neutrophils in the skin following topical application of a skin irritant. The α7KO mice, independent of the mouse strain backgrounds tested, have elevated inflammatory cytokines (IL-1β and IL-6, but not necessarily TNFα) and increased expression of adhesion molecules and chemokines that are important to recruitment of the pro-inflammatory cells to the skin. One challenge remaining will be to determine if such regulatory interactions among nAChR and inflammatory systems are equally customized to the microenvironment of other organs such as gut, secondary lymphoid organs, as well as the brain.

## Abbreviations

nAChR: neuronal nicotinic acetylcholine receptor; IL: interleukin; TNF: tumor necrosis factor; qPCR: quantitative PCR.

## Competing interests

The authors declare that they have no competing interests.

## Authors' contributions

LCG and SWR designed the experiments, analyzed the data, and wrote the manuscript.

AVO initiated the study, implemented techniques, analyzed data, and contributed to the writing. MR contributed technical expertise and analysis of data as well as provided editing of the manuscript. All of the authors have read and approved the final version of the manuscript.
